# Transcriptomic Insights into the Enhanced Aroma of Guangdong Oolong Dry Tea (*Camellia sinensis* cv. Yashixiang Dancong) in Winter

**DOI:** 10.3390/foods13010160

**Published:** 2024-01-02

**Authors:** Yanchun Zheng, Peifen Chen, Peng Zheng, Jiahao Chen, Binmei Sun, Shaoqun Liu

**Affiliations:** College of Horticulture, South China Agricultural University, Guangzhou 510642, China; zhengyanchun@stu.scau.edu.cn (Y.Z.); 13729290220@163.com (P.C.); zhengp@scau.edu.cn (P.Z.); cjhtea@stu.scau.edu.cn (J.C.); binmei@scau.edu.cn (B.S.)

**Keywords:** multi-omics analysis, Guangdong oolong tea, aroma formation, winter

## Abstract

Winter dry tea (WDT) exhibits a more intense and lasting aroma compared to dry tea from other seasons; however, this conclusion is solely based on sensory outcomes and lacks corroborative theoretical evidence. Our study aimed to analyze the aroma compounds in WDT and investigate the causes behind the formation of WDT’s aroma by analyzing the volatile organic compounds (VOCs) in WDT, spring dry tea (SDT), winter fresh leaves (WFLs) and spring fresh leaves (SFLs) by gas chromatography-mass spectrometry (GC-MS), complemented by an analysis of gene expression pertinent to WFLs and SFLs by using transcriptomic analysis. The results revealed a significant increase in total VOCs in WDT compared to SDT, with WDT exhibiting distinct woody aromas as indicated by a higher *α*-muurolene content. In WFL, the contents of aldehydes and ketones were richer than those in SFL. Notably, the study found that UDP-glycosyltransferase genes in WFLs were significantly up-regulated, potentially promoting the synthesis of terpene glycosides. These terpene glycosides can release terpene aroma compounds during processing, contributing significantly to the intense and lasting aroma of WDT. Overall, this research provides valuable insights into the mechanism behind aroma formation in Guangdong oolong tea harvested during winter.

## 1. Introduction

Tea (*Camellia sinensis* L.) is the most popular non-alcoholic beverage, consumed worldwide for its charming flavor. Based on its processing methods, tea is categorized into three main types—green tea, black tea and oolong tea—each possessing distinct flavor characteristics. Oolong tea, in particular, is renowned for its elegant fruity and floral notes [1]. Some famous oolong teas include Tieguanyin from Anxi in Fujian Province, Wuyi rock tea from Wuyi Mountain in Fujian Province, Dongding oolong tea from Taiwan and Fenghuang Dancong (FHDC) from Chaozhou in Guangdong Province.

FHDC stands out for its unique aroma and diverse fragrance profiles, which are shaped by factors such as its cultivar, manufacturing processes and environmental conditions [2,3,4]. Harsh conditions like low humidity, intense light and dry heat in summer and autumn often degrade the quality of tea leaves [1,5], while the mild temperatures and high humidity of spring enhance it. In the birthplace of FHDC (Chaozhou, China,), the moderate winter temperatures from 10 °C to 20 °C support tea plants’ normal physiological activities. FHDC produced in this season exhibits a more intense and lasting aroma, potentially linked to the lower ambient temperature [6]. However, the mechanism behind the enhanced aroma of FHDC produced in winter remains largely unexplored.

The aroma of tea mainly stems from volatile terpenes, esters, carotenoids, phenylpropane compounds and their derivatives [7,8,9]. Oolong tea is rich in terpene aroma compounds, with monoterpenes and sesquiterpenes, such as linalool and its oxides, *α*-phellandrene, geraniol and (*E*)-nerolidol, being the primary contributors to its fragrance [10,11]. 

Terpenoid biosynthesis primarily occurs through the mevalonate (MVA) and methylerythritol phosphate (MEP) pathways. The MEP pathway, occurring in plastids, facilitates the synthesis of monoterpenes and diterpenes [12]. In contrast, the MVA pathway, located in the cytoplasm, is responsible for producing sesquiterpenes, sterols and triterpenes [13,14]. These pathways function independently but exhibit metabolic crosstalk [15]. The MEP and MVA pathways have distinct sets of enzyme-catalyzed reactions, seven for MEP and six for MVA [16], both of which lead to the production of the precursors isopentenyl pyrophosphate (IPP) and dimethylallyl pyrophosphate (DMAPP) [16]. These precursors, under the influence of terpene synthases, undergo rearrangement and cyclization reactions to produce various terpenoid compounds [17,18]. 

Cold environments stimulate enhanced synthesis and emissions of large quantities of terpene compounds such as linalool and nerolidol, supporting the cold tolerance of tea plants [19]. Tea processing methods, especially those involving external pressure, further amplify the release of these aroma compounds [1]. Furthermore, the action of UDP-glycosyltransferases (UGTs) enables terpenoid aroma compounds to combine with sugar molecules, forming glycosides [20]. Although tasteless, these glycosides are hydrolyzed into aromatic volatile compounds by glycosidases [21].

Since the fresh leaves of tea trees in spring are the main raw material used to process dry tea, in this study, we analyzed the aroma of fresh FHDC leaves in both the spring and winter seasons, incorporating transcriptome sequencing to unveil the underlying formation mechanisms of aroma development. Our findings may offer insights into the aromatic characteristics of winter-harvested Guangdong oolong tea.

## 2. Materials and Methods

### 2.1. Materials

Materials were collected from five-year-old ‘Yashixiang Dancong’ (YD) tea plants in Guangdong Province (23.93° N, 116.70° E). In the winter of October 2021 (14 °C, 1007 hPa, relative humidity (RH) 73%) and in the spring of April 2022 (22 °C, 996 hPa, RH 88%), the first two leaves and buds were plucked during collection. With respect to each season’s treatment, six replicates were conducted. All samples for this experiment were collected from the same tea garden (2000 m^2^). The amount harvested for each replicate was 30 kg. The same oolong processing technique was used to process fresh leaves (FLs) from spring and winter into dry tea (DT). Spring fresh leaves (SFLs) and winter fresh leaves (WFLs) were immediately transferred to liquid nitrogen before storage at −80 °C for further analysis. Spring dry tea (SDT) and winter dry tea (WDT) were stored in airtight canisters.

### 2.2. Extraction of the Volatile Components

Headspace solid-phase microextraction (HS-SPME) was used to extract the volatile components of tea leaves [2]. The DT and FLs were ground into a fine powder in a mortar and pestle using liquid nitrogen. Then, 2 g of DT powder, 5 mL of saturated sodium chloride solution and 0.0864 g of ethyl decanoate were added to an HS vial, which was then sealed quickly. For the fresh leaves, 0.2 g of FL powder, 5 mL of saturated sodium chloride solution and 0.0864 g of ethyl decanoate were added to another HS vial, which was then sealed quickly. Divinylbenzene/carboxyl/polydimethylsiloxane (DVB/CAR/PDMS) SPME fibers (50/30 µm inner diameter, 2 cm long) were preheated at 80 °C for 15 min, then inserted into the HS vial for a 40 min extraction. The SPME fibers with adsorbed tea volatile components were inserted into the GC inlet at 250 °C for 3 min for enhanced resolution. Six replicates were performed for each sample.

### 2.3. GC-MS Analysis

A GC-MS analysis was performed on an 1890B gas chromatograph (Agilent, Santa Clara, CA, USA) equipped with a 5977A mass spectrometer. The chromatographic column was an HP-5MS capillary column (30 m × 0.25 mm × 0.25 um). The column flow rate was 1.0 mL/min with pure nitrogen (99.999% purity) as the carrier gas in split mode. The instrument was maintained at an initial temperature of 50 °C for 1 min, then ramped up to 220 °C at a rate of 5 °C/min for 5 min. The MS conditions were as follows: the ion source temperature was 230 °C, the electron energy was 70 eV, the scan range was 30–400 amu and the solvent delay time was 4 min.

### 2.4. GC-MS Data Analysis

The volatile components of tea aroma were identified by the retention index (RI) and mass spectrometry matching methods [22,23]. The n-alkane standards C9–C21 were subjected to a GC-MS analysis under the same ramp-up procedure to obtain their retention times. The RI was calculated as follows:

$$RI=100n+100\times \frac{RT(x)-RT(n)}{RT(n+1)-RT(n)}$$

where RT(x) is the retention time of volatile component x, and RT(n) and RT(n + 1) are the retention times of the alkanes of C(n) and C(n + 1) that immediately preceded and followed the elution of volatile component x.

The quantification of volatile components was performed using peak areas of internal standards and their concentrations:

$$C_{i}=\frac{S_{i}}{S_{s}}\times \frac{m_{s}}{m_{}}$$

where C_i_ is the absolute concentration (ng/g) of volatile component i, S_i_ is the peak area of volatile component i, S_s_ is the peak area of the internal standard, m is the mass in g of the tea sample and m_s_ is the mass in ng of the internal standard.

### 2.5. Calculation of Odor Activity Value (OAV) and Terpene Index (TI)

The OAV is frequently used to estimate the contribution of aroma components to the overall aroma. The OAV is defined as the ratio between the odor threshold (OT) of an aroma component in water and the concentration of the corresponding aroma component:

$$OAV=\frac{C_{i}}{T_{i}}$$

where C_i_ is the concentration of volatile i (ng/g) and T_i_ is the OT of compound i (ng/g) [24]. Aroma substances contribute to the tea aroma when 0 ≤ OAV < 1, and they make a significant contribution when OAV ≥ 1 [25]. 

The TI is commonly used to evaluate the aroma characteristics of tea. The calculation method for the TI was as follows:

$$TI=\frac{S_{l}}{S_{l}+S_{g}}$$

where S_l_ is the peak area of linalool, and S_g_ is the peak area of geraniol [26]. The tea has a rich and pleasant aroma when the TI is high, and the tea has a strong and sharp fragrance when the TI is low [26]. 

### 2.6. RNA Extraction and Transcriptome Sequencing

WFLs and SFLs were used for transcriptome sequencing, and three biological replicates were performed. The samples were ground to powder with liquid nitrogen. RNA was extracted according to the instructions of the kit (hipure plant RNA mini kit B, R4151-02B, Magen Biotechnology Co., Ltd., Guangzhou, China). RNA concentration and integrity were assessed on 2% agarose gels and with a 2100 Bioanalyzer (Agilent, Santa Clara, CA, USA). The RNA samples were sent to Genedenovo Biotechnology Co., Ltd. (Guangzhou, China) for cDNA library construction and sequenced on an Illumina sequencing platform (Illumina, New England Biosciences, Santa Clara, CA, USA).

### 2.7. Transcriptome Analysis

The quality control of transcriptome raw data was performed using fastp to filter out low-quality data [27]. The short-read matching tool bowtie2 was used to align the clean reads against the ribosome database and remove unmapped reads [28]. Global and local alignment searches were performed using HISAT2 to match spliced reads in RNA Seq data [29]. The fragments per kilobase of exon model per million mapped fragments (FPKM) method was applied to calculate gene expression levels [30,31]. The differentially expressed genes (DEGs) were annotated with gene ontology (GO) terms, and the number of genes associated with each GO function was determined. The hypergeometric test was applied to identify Kyoto Encyclopedia of Genes and Genomes (KEGG) pathways that showed significant enrichment among the DEGs.

### 2.8. qRT-PCR 

The sequences used to design qRT-PCR are listed in the Supplementary Table S1. qRT-PCR was performed with a LC480 PCR (Roche, Basel, Switzerland) according to our previously published study [32]. Actin was used as the internal reference, and relative expression levels were calculated using the 2^−ΔΔCT^ method. 

### 2.9. Statistical Analysis

Coefficients of variation (CV%) and *t*-tests followed by a Mann–Whitney test were calculated using Excel 2020 and GraphPad Prism 9.0 to assess statistical significance. A principal component analysis (PCA) of volatile components was performed using GraphPad Prism 9.0. The values are shown as mean ± SD (*n* = 3).

## 3. Results

### 3.1. Volatile Components of DT

**Figure 1:**
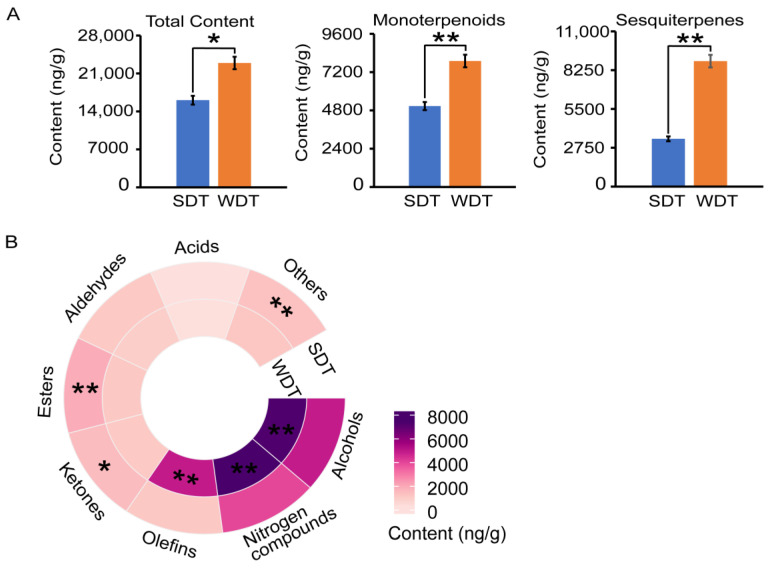
Aroma analysis of spring dry tea (SDT) and winter dry tea (WDT). (**A**) Concentrations of the total aroma compounds, monoterpenoids and sesquiterpenes in SDT and WDT. (**B**) The concentrations of substance categories in SDT and WDT. Student’s *t*-test was used to identify significant differences (* *p* < 0.05; ** *p* < 0.01).

The aroma qualities of SDT and WDT were determined by analyzing their volatile components using GC-MS (Figure S1A). The total concentration of volatiles in WDT was 22,930 ng/g, whereas that of SDT was 16,080 ng/g (Figure 1A, Table S3). The TI of SDT was 0.72, whereas that of WDT was 0.87 (Table S4). Both the total contents of monoterpene aroma compounds and sesquiterpene aroma compounds in WDT were significantly greater than those in SDT (Figure 1A). In addition, the contents of alcohols, nitrogen compounds and olefins in WDT were significantly higher than those in SDT (Figure 1B). In contrast, the contents of ketones, esters and other volatile compounds were significantly higher in SDT than WDT.

### 3.2. Quality Analysis of DT

**Figure 2:**
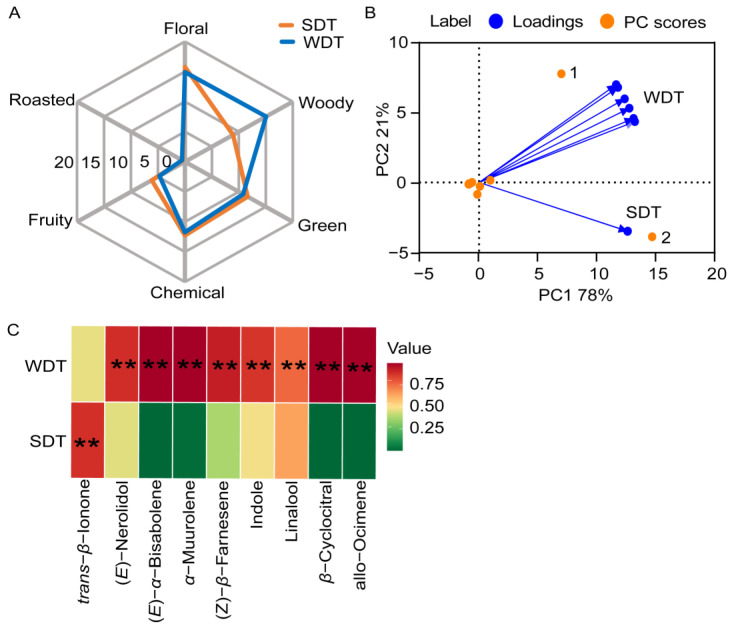
Aroma analysis of dry tea (DT). (**A**) Aroma radar map of spring dry tea (SDT) and winter dry tea (WDT). (**B**) Principal component analysis (PCA) of volatile components of SDT and WDT. (**C**) The content of important aroma compounds in SDT and WDT. Student’s *t*-test was used to identify significant differences (** *p* < 0.01).

Among the volatile components of SDT and WDT, 19 contributed to floral aromas, three to fruity aromas and three to green aromas (Table S5). The total OAVs for each aroma dimension were logarithmically transformed to generate an aroma radar plot (Figure 2A). The DTs exhibited pronounced floral aromas during both seasons. Roasted aromas were not prominent in either SDT or WDT. Green, chemical and fruity aromas played secondary roles in the overall aromas of SDT and WDT. Notably, WDT exhibited distinct woody aromas, while SDT had a more prominent green and fruity aroma. These aroma results align with the characteristic aromas of SDT and WDT.

To gain a further understanding of the aroma profile of DT, a PCA was conducted on 31 aroma volatiles from SDT and WDT (Figure 2B). We also analyzed the content and OAV of significant aroma compounds (Figure 2C, Table 1). The PCA results revealed that the first principal component (PC1) accounted for 21% of the total variance, while PC2 explained 78%. Notably, the analysis revealed *α*-muurolene (point 1) as the characteristic aroma compound for WDT, while *trans*-*β*-ionone (point 2) was the characteristic aroma compound for SDT. These specific compounds are key to differentiating the aromatic profiles of SDT and WDT. The content of *α*-muurolene in WDT was significantly higher than that in SDT, and the content of *trans*-*β*-ionone in SDT was significantly higher than that in WDT. Upon examining other aroma compounds, WDT had significantly higher levels of (*E*)-nerolidol, (*Z*)-*β*-farnesene, indole, linalool, (*E*)-*α*-bisabolene, allo-ocimene, *β*-cyclocitral and *α*-muurolene compared to SDT. In terms of the OAV, WDT surpassed SDT for (*E*)-nerolidol, (*Z*)-*β*-farnesene, indole, linalool and *α*-muurolene; however, WDT had a lower OAV for *trans*-*β*-ionone. Specifically, the OAV of *α*-muurolene was 439.50 in SDT and 33,902.70 in WDT. On the other hand, the OAV of *trans*-*β*-ionone in WDT was 25,580.00, while it reached 48,204.00 in SDT.

### 3.3. Volatile Components of FLs

**Figure 3:**
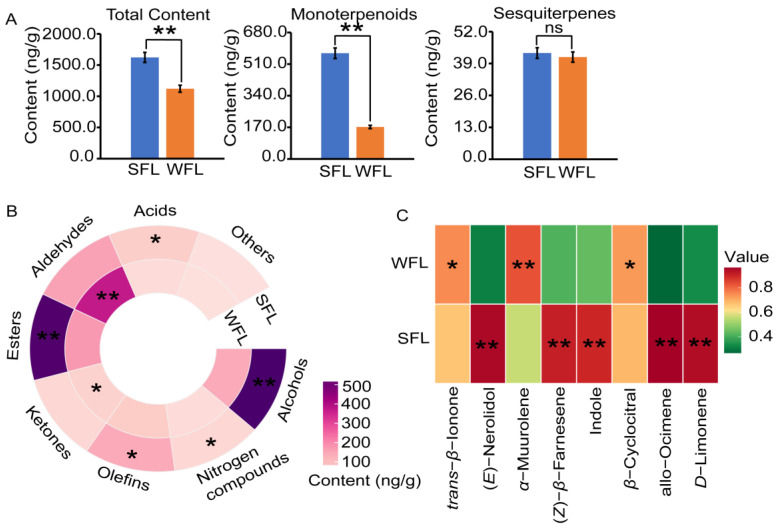
Aroma analysis of fresh leaves (FLs). (**A**) Concentrations of total aroma compounds, monoterpenoids and sesquiterpenes in spring FLs (SFL) and winter FLs (WFL). (**B**) The contents of substances in various chemical categories in SFLs and WFLs. (**C**) The concentrations of important aroma components in SFLs and WFLs. Student’s *t*-test was used to identify significant differences (* *p* < 0.05; ** *p* < 0.01; ns indicates no significant difference).

Next, we utilized GC-MS to examine the aroma components of SFLs and WFLs (Figure S1B). Overall, the total concentration of volatile components in the SFLs, at 1622.20 ng/g, was notably greater than that in the WFLs, which was 1120.32 ng/g (Figure 3A, Table S1). Despite this difference, the content of sesquiterpene aroma compounds in the two seasons was comparable. The TIs of the SFLs and WFLs were 0.31 and 0.00, respectively (Table S4). The total amount of monoterpene aroma compounds in the SFLs was significantly higher than that in the WFLs (Figure 3A). Additionally, the WFLs displayed significantly higher levels of ketones and aldehydes compared to the SFLs (Figure 3B).

For a more detailed comparison between the SFLs and WFLs, we focused on eight important aroma compounds (Figure 3C). Specifically, the contents of (*E*)-nerolidol, (*Z*)-*β*-farnesene, indole, *D*-limonene and allo-ocimene were considerably higher in the SFLs than in the WFLs. Conversely, the WFLs had a notably higher *α*-muurolene content than the SFLs. Similarly, the WFLs contained significantly more *β*-cyclocitral and *trans*-*β*-ionone compared to the SFLs.

### 3.4. Screening and Analysis of DEGs

**Figure 4:**
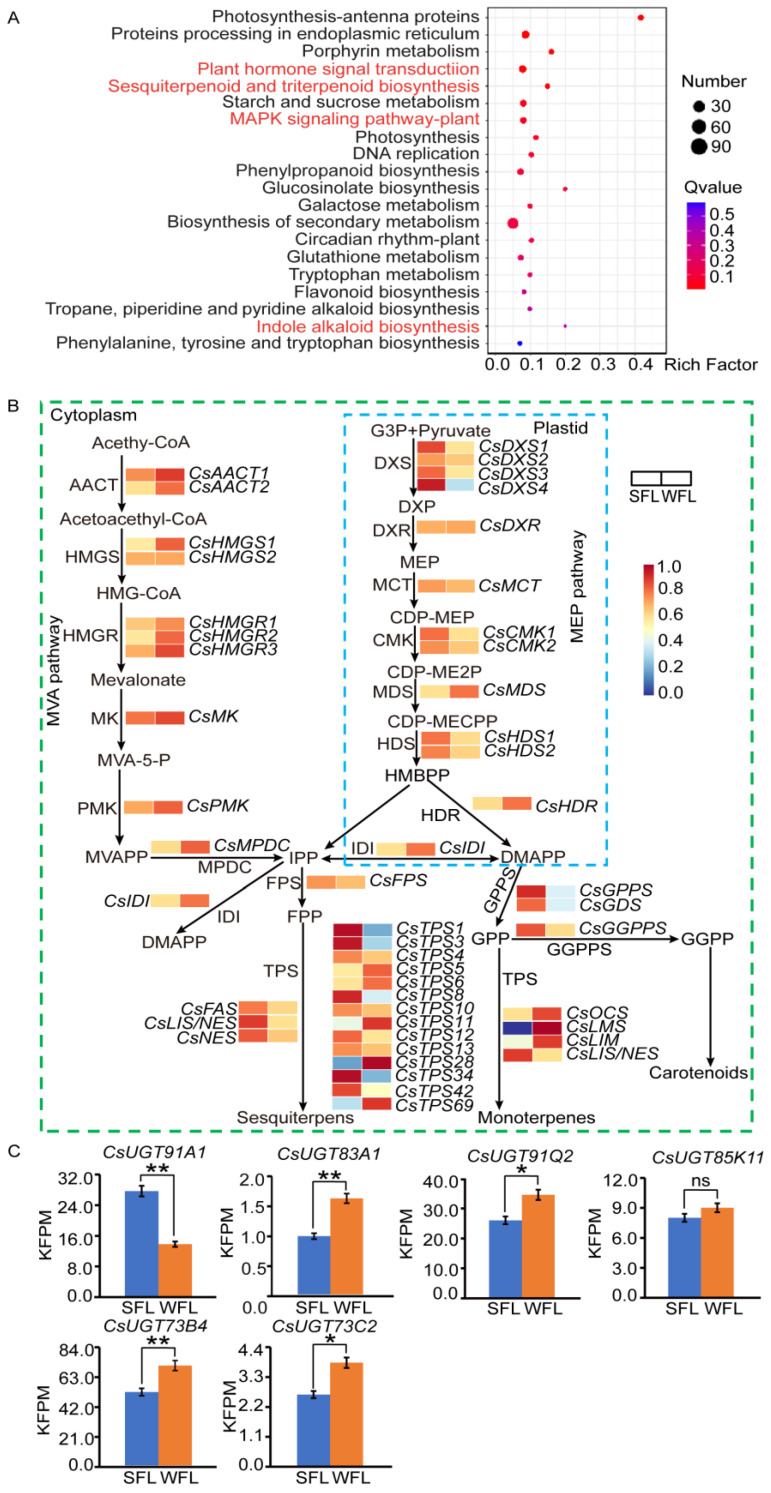
Analysis of differentially expressed genes (DEGs) in spring (SFL) and winter fresh leaves (WFL). (**A**) Compared to SFL, DEGs of WFLs exhibited distinct enrichment within KEGG pathways. (**B**) The terpene biosynthesis pathway, with relative expression of related genes in WFLs vs. SFLs. Abbreviations: AACT, acetoacetyl-*CoA* thiolase; HMGS, 3-hydroxy-3-methylglutaryl synthase; HMGR, 3-hydroxy-3-methylglutaryl-*CoA* reductase; MK, mevalonate kinase; PMK, phosphomevalonate kinase; MPDC, mevalonate diphosphate decarboxylase; DXP, 1-deoxy-*D*-xylulose 5-phosphate; DXS, DXP synthase; DXR, 1-deoxy-*D*-xylulose 5-phosphate reductoisomerase; MCT, 2-*C*-methyl-*D*-erythritol 4-phosphate cytidylyltransferase; CMK, 4-(cytidine 5′-diphospho)-2-*C*-methyl-*D*-erythritol kinase; MDS, 2-*C*-methyl-*D*-erythritol 2,4-cyclodiphosphate synthase; HDS, 4-hydroxy-3-methylbut-2-enyldiphosphate synthase; HDR, 1-hydroxy-2-methyl-2-(*E*)-butenyl-4-diphosphate reductase; IDI, isopentenyl pyrophosphate isomerase; FPP, farnesyl pyrophosphate; FPS, FPP synthase; GGPP, geranylgeranyl pyrophosphate; GGPPS, GGPP synthase; LMS, limonene synthase; GPP, geranyl pyrophosphate; GPPS, GPP synthase; IPP, isopentenyl pyrophosphate; DMAPP, dimethylallyl pyrophosphate; TPS, terpene synthase; FAS, farnesene synthase; AFS, *α*-farnesene synthase; NES, nerolidol synthase; LIS/NES, linalool/nerolidol synthase; LIM, limonene synthase; OCS, ocimene synthase; GDS, geranylgeranyl diphosphate synthase. (**C**) Expression levels of *CsUGT91A1*, *CsUGT83A1*, *CsUGT91Q2*, *CsUGT85K11*, *CsUGT73B4* and *CsUGT73C2* in SFLs and WFLs. Student’s *t*-test was used to identify significant differences (* *p* < 0.05; ** *p* < 0.01; ns indicates no significant difference).

To investigate the regulatory mechanism of terpenoid synthesis, we conducted transcriptome sequencing of the WFLs and SFLs. A total of 1971 DEGs were identified in the WFLs, with 1338 up- and 633 down-regulated, relative to the SLFs (Figure S3). These DEGs were enriched in KEGG pathways associated with the tea tree terpene synthesis pathway, including “plant hormone signal transduction”, “sesquiterpenoid and triterpenoid biosynthesis”, “MAPK signaling pathway-plant” and “indole alkaloid biosynthesis” (Figure 4A). 

Because triterpenoids make a great contribution to Oolong tea’s aroma, we focused on the DEGs in that pathway. In all, 47 genes related to terpene synthesis were analyzed (Figure 4B). During winter, the majority of the genes in the MVA pathway were up-regulated, while MEP pathway genes were largely down-regulated. *CsIDI*, which facilitates the interconversion of IPP and DMAPP, was up-regulated during the winter season. Furthermore, genes involved in limonene synthesis, namely *CsLMS* and *CsLIM*, and the ocimene synthase gene, *CsOCS*, exhibited up-regulation during winter. Four terpene synthesis genes (*CsTPS5, CsTPS6, CsTPS11* and *CsTPS28*) also had increased expression in winter. However, some terpene synthase genes, such as farnesene synthase (*CsFAS*) and nerolidol synthase (*CsNES, CsLIS/NES*), were down-regulated during winter. Additionally, five UDP-glycosyltransferase genes (*CsUGT91Q2*, *CsUGT85K11*, *CsUGT73B4*, *CsUGT73C2* and *CsUGT83A1*) exhibited elevated expression levels in winter relative to spring. On the other hand, *CsUGT91A1* exhibited lower expression levels in winter compared to spring (Figure 4C). 

### 3.5. Quantitative RT-PCR Analysis

**Figure 5:**
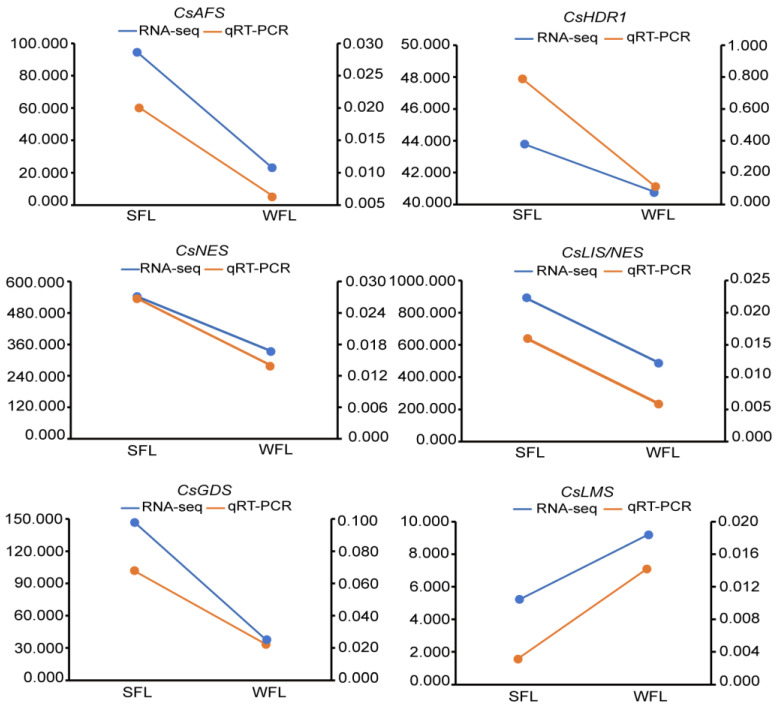
Comparison of six random unigenes’ expression via quantitative real-time PCR (qRT-PCR) with transcriptome sequencing results. The left *Y*-axis represents the qRT-PCR results, and the right *Y*-axis represents the transcriptome sequencing results. AFS, alpha-farnesene synthase; HDR, 1-hydroxy-2-methyl-2-(*E*)-butenyl-4-diphosphate reductase; NES, nerolidol synthase; LIS/NES, linalool/nerolidol synthase; GDS, geranylgeranyl diphosphate synthase; LMS, limonene synthase.

In order to evaluate the accuracy of the transcriptome data, we randomly selected six unigenes from the DEGs for a gene expression analysis by qRT-PCR. The results of the six unigenes were consistent with the transcriptome sequencing results in that their expression levels followed the same trends (Figure 5).

## 4. Discussion

### 4.1. Superior Aroma Profile of WDT over SDT

On Fenghuang Mountain, tea plants maintain their physiological activities during winter, which forms the basis for WDT’s pleasing aroma. Though tea processing significantly influences aroma enhancement, this study revealed that WDT’s OAV surpassed that of SDT, despite undergoing the same processing regime (Table S5). Both SDT and WDT had higher levels of aroma compounds relative to their fresh leaves. Despite SFLs having higher aroma compound concentrations than WFLs, WDT surpassed SDT in this regard. Under uniform processing conditions, WDT synthesized additional aroma compounds, particularly nerolidol, farnesene, indole, linalool and defensive aroma compounds (e.g., (*E*)-*α*-bisabolene, allo-ocimene and *β*-cyclocitral). Given that the SFLs and WFLs underwent the same processing, colder temperatures likely augmented WDT’s aroma intensity and persistence. Consequently, harvesting FLs at colder temperatures not only elevates the aroma content of DT but also boosts its overall OAV.

### 4.2. Low Temperatures Influence the Aroma of DT

The aroma profile of tea is primarily determined by specific aroma constituents rather than the total concentration of volatile compounds [35,36]. In this study, we explored this complexity, drawing on odor descriptions (ODs) and OTs from earlier research (Table S5). Under identical processing conditions, SDT and WDT displayed distinct aroma profiles (Figure 2A). SDT was characterized by a floral aroma due to the presence of *trans*-*β*-ionone, linalool and (*E*)-nerolidol. Conversely, WDT combined floral and woody scents, with higher OAVs of linalool and (*E*)-nerolidol contributing to its floral aroma and *α*-muurolene to its woody scent. *α*-Muurolene, which has a pronounced woody aroma and a low threshold in water, is found in higher concentrations in WDT [24]. This makes it an important contributor to the tea’s aroma profile. We hypothesize that processing tea leaves under low-temperature conditions may be conducive to the biosynthesis of α-Muurolene, or alternatively, that fresh leaves may have already synthesized a higher amount of precursors to α-Muurolene in a low-temperature environment. We plan to investigate this hypothesis in future research. In conclusion, the aroma characteristics of DT can be influenced by the environmental factors experienced by the plant, with low-temperature environments amplifying the role of *α*-muurolene in defining WDT’s aroma.

### 4.3. Multiple Biological Pathways Involved in Regulating the Formation of Terpene Aroma Compounds in WDT

A KEGG enrichment analysis of WFLs’ DEGs revealed prominent pathways including “plant hormone signal transduction”, “sesquiterpenoid and triterpenoid biosynthesis”, “MAPK signaling pathway-plant” and “indole alkaloid biosynthesis”. It has been reported that in low-temperature conditions, tea plants absorb nerolidol from their surroundings. This nerolidol is then transformed into bound nerolidol glucoside via the action of UGT enzymes [20]. The presence of nerolidol in tea leaves triggers the activation of mitogen-activated protein kinases (MAPKs) and the production of plant hormones [22]. This is significant because hormone signaling mediates the response of *CsTPS* to external stresses [37]. Zhu et al. [38] further established that these plant hormones amplify the biosynthesis of terpenoid compounds. Therefore, we hypothesize that under low-temperature conditions, tea plants may absorb terpenoids from the surrounding environment. These terpenoids could activate MAPKs and induce the production of plant hormones, which are beneficial for the biosynthesis of terpenoids. Furthermore, during the winter’s low temperatures, terpenoids are likely converted into their corresponding glycosides through the action of UGT enzymes. These synthesized terpenoid glycosides have a high potential to transform into volatile terpenoid aroma compounds during the processing of dry tea. 

Tea leaves are subjected to various external stresses during processing. It has been reported that under the dual influence of low temperatures and mechanical damage, tea leaves can respond by synthesizing more indole and nerolidol [39,40]. The content of indole and nerolidol in SDT increased by 3001.08 ng/g and 2182.23 ng/g, respectively, while the content of indole and nerolidol in WDT increased by 5444.03 ng/g and 4267.67 ng/g, respectively (Table S3). Interestingly, after processing, defensive compounds such as allo-ocimene, *β*-cyclocitral and (*E*)-*α*-bisabolene were only detected in WDT (Table S3) [41,42,43]. It is speculated that external stresses during processing stimulated other metabolic processes involved in aroma synthesis, thereby enhancing the aroma of WDT. 

### 4.4. Expression of Key Genes Regulates Terpene Aroma Synthesis

The gene expression levels in the MVA and MEP pathways are inconsistent between the SFLs and WFLs, yet the WFLs synthesized fewer monoterpenes and sesquiterpenes than the SFLs. This discrepancy suggests that these pathways may predominantly affect the formation of intermediate compounds in FL aroma synthesis, a process potentially affected by the interconversion between IPP and DMAPP, which could lead to cross-talk between the MVA and MEP pathways. Interestingly, the winter expression levels of *CsLMS* and *CsLIM* were higher than in spring, but the WFLs had a lower limonene content than the SFLs. This observation supports the notion that terpene biosynthesis is a complex process influenced by multiple genes rather than being determined by the expression of a single gene. 

The increased expression level of *CsUGT* in the WFLs compared to the SFLs highlights the significant role of UGTs in converting free terpenes into glycosides. We speculate that under low temperatures, the WFLs synthesized more terpene aroma glycosides, resulting in a reduced aroma content compared to the SFLs. During processing, these glycosides were then converted into terpene aroma compounds, enhancing the aroma content of WDT. 

Zhao et al. [20] established a positive correlation between the content of nerolidol glycosides and *CsUGT91Q2*, and Ohgami et al. [21] demonstrated the broad substrate specificity of *CsUGT85K11* towards a variety of aroma compounds. These findings are crucial for understanding transformations during tea processing. Under various stresses, FL tissues facilitate the conversion of terpene aroma glycosides into free aroma compounds through the action of *β*-primeverosidase [35]. In this study, we found that the WFLs had lower levels of terpene aroma compounds compared to the SFLs, a trend that is especially pronounced in the nerolidol and indole content of WDT compared to the SFLs. Consequently, the distinct aroma of WDT could be associated with the expression of *CsUGT* in WFLs, suggesting a future research direction to explore the specific role of *CsUGT* in shaping the aroma profile in winter tea leaves. 

## 5. Conclusions

In conclusion, low temperatures significantly contribute to the distinct aromas observed in SDT and WDT. A combination of biological processes, particularly the dual effects of low temperatures and mechanical damage during processing, enhanced the synthesis of aroma compounds and boosted their OAVs in WDT. The analysis of the FL transcriptome suggested a correlation between the aroma profile of WDT and the expression levels of *CsUGT* in FLs under cooler conditions. 

## Figures and Tables

**Table 1:** The odor activity value (OAV) of key volatile components of spring dry tea (SDT) and winter dry tea (WDT).

Volatile Compounds	Odor Type	Odor Threshold (ng/g)	OAV ^d^	
			SDT	WDT
(*E*)-Nerolidol	Floral	10.00 ^c^	219.08 ± 35.33	427.05 ± 71.72
(*Z*)-*β*-Farnesene	Floral	87.00 ^a^	1.25 ± 0.17	3.07 ± 0.50
Indole	Floral	40.00 ^a^	75.51 ± 8.96	136.34 ± 10.74
Linalool	Floral	0.22 ^a^	3887.77 ± 660.68	4616.14 ± 762.64
*trans*-*β*-Ionone	Floral	0.01 ^a^	48,204.00 ± 7662.00	25,580.00 ± 4925.00
*α*-Muurolene	Woody	0.10 ^b^	439.50 ± 65.80	33,902.70 ± 2223.10

Odor thresholds were from ^a^ [22], ^b^ [33] and ^c^ [34]. ^d^ Values are the mean ± standard deviation of three replicates.

## Data Availability

Data are contained within the article.

## Supplementary Materials

The following supporting information can be downloaded at https://www.mdpi.com/article/10.3390/foods13010160/s1. Figure S1: GC-MS spectra of compounds in DT and FL; Figure S2: Agarose gel electrophoresis of RNA from SFL and WFL; Figure S3: Differentially expressed genes in SFL and WFL; Table S1: Primer table for fluorescence quantitative PCR validation; Table S2: Quality of reads for 6 RNA-Seq samples; Table S3: The volatile components of FYS, FYW, YDS and YDW; Table S4: Terpene index of SFL, WFL, SDT and WDT; Table S5: The OAV of volatile components in SDT and WDT. References [44,45] is cited in Supplementary Materials.
