# Supplementary material for: Transcriptomic Insights into the Enhanced Aroma of Guangdong Oolong Dry Tea (Camellia sinensis cv. Yashixiang Dancong) in Winter
Source: Foods. 2024 Jan 2;13(1):160. doi: 10.3390/foods13010160 (PMC10778534; doi:10.3390/foods13010160)
Supplement: Supplementary file 1 [file foods-13-00160-s001.zip › foods-2791594-supplementary.pdf]

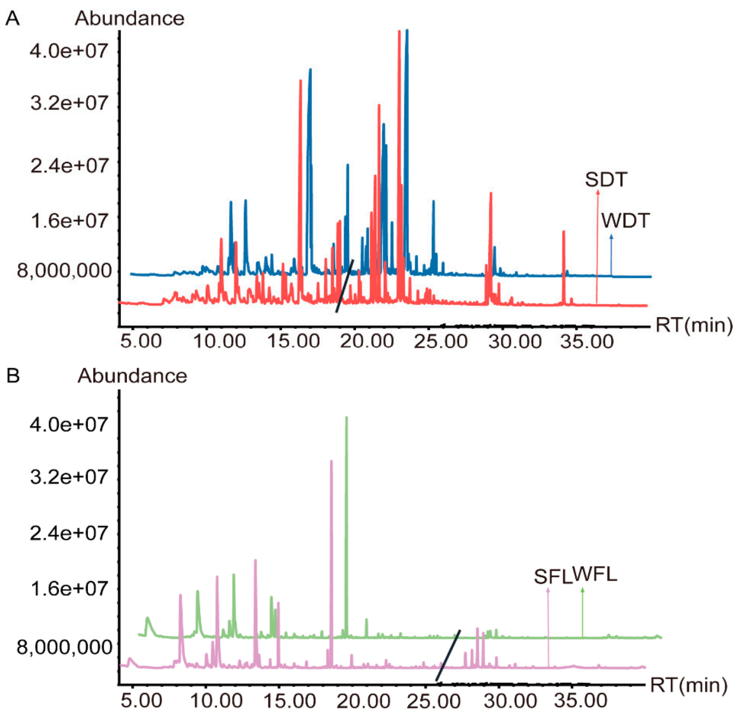

Fig. S1. GC-MS spectra of compounds in DT and FL. (A) GC-MS spectra of compounds in SDT and WDT. (B) GC-MS spectra of compounds in SFL and WFL.

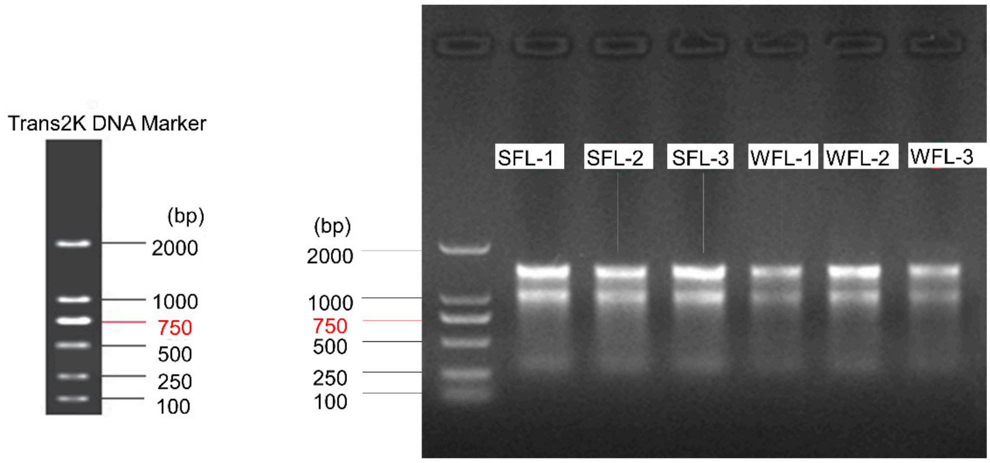

Fig. S2. Agarose gel electrophoresis of RNA from SFL and WFL.

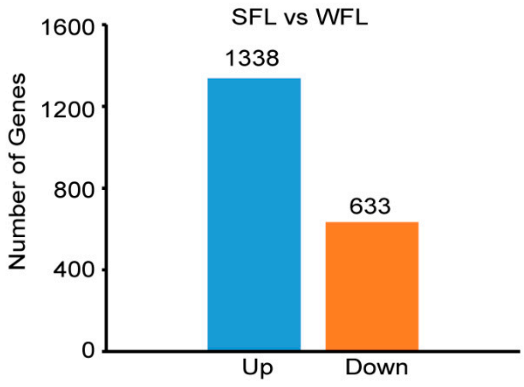

Fig. S3. Differentially expressed genes in SFL and WFL.

Table S1. Primer table for fluorescence quantitative PCR validation.

Unigene ID	Primer sequences（5’-3’）	Primer length（bp）
CsActin	F: GCCATATTTGATTGGAATGG	20
	R: GGTGCCACAACCTTGATCTT	20
CsLMS	F: GTTGACAGATGGGATGTG	18
	R: CAATGTTGGCTTGTACCC	18
CsGDS	F: GGTACCGGAGGTCACTCG	18
	R: TGTCATCATGGTGATCAT	18
CsLIS/NES	F: GCAAGAAGGGTACAATGTGCCCGCG	25
	R: CTGCGGCTTGATCAAGAATGTCTTC	25
CsNES	F: AGGCATTGATTACCACTTCCGGGAG	25
	R: GCACATTGTAGCCTTGTTGCCTCAA	25
CsHDR1	F: GGGGTTTGATCCAGACAG	18
	R: TGAAGTGCTCGTTGAGGT	18
CsAFS	F: TGTCAACACAGCTAGAGTGGC	21
	R: AGCATAGAGAGGACTTGGGC	21

Table S2. Quality of reads for 6 RNA-Seq samples.

Sample	RawData (bp)	CleanData (bp)	Q20 (%)	Q30 (%)	N (%)	GC (%)
**SFL-1**	7,983,845,400	7,922,126,134	7,749,669,496 (97.82%)	7,420,775,626 (93.67%)	45,172 (0.00%)	3,580,115,679 (45.19%)
**SFL-2**	8,568,089,700	8,496,049,913	8,318,335,734 (97.91%)	7,976,296,814 (93.88%)	48,509 (0.00%)	3,852,622,084 (45.35%)
**SFL-3**	8,621,056,200	8,545,617,746	8,371,429,568 (97.96%)	8,033,166,400 (94.00%)	48,787 (0.00%)	3,857,775,196 (45.14%)
**WFL-1**	8,386,216,500	8,323,360,731	8,164,355,999 (98.09%)	7,846,314,729 (94.27%)	47,005 (0.00%)	3,702,275,001 (44.48%)
**WFL-2**	7,352,164,800	7,297,175,748	7,145,505,785 (97.92%)	6,849,630,299 (93.87%)	31,880 (0.00%)	3,261,275,334 (44.69%)
**WFL-3**	8,146,940,700	8,071,044,551	7,894,668,388 (97.81%)	7,559,522,773 (93.66%)	30,662 (0.00%)	3,674,170,692 (45.52%)

Table S3. The volatile components of FYS, FYW, YDS and YDW.

No.	Volatile Compounds	SFL (ng/g)	WFL (ng/g)	SDT (ng/g)	WDT (ng/g)	RI (cal)/RI (ref)	Qualitative method
1	Benzyl alcohol	26.28±3.52	-	229.9±62.75	270.91±23.67	1030/1034	MS, RI
2	Linalool	75.53±45.7	-	855.31±145.35	1015.55±167.78	1099/1098	MS, RI
3	Linalool oxide I	94.21±20.47	40.21±5.55	375.56±58.16	190.85±21.95	1069/1071	MS, RI
4	Linalool oxide II	170.41±39.04	77.39±11.90	96.29±17.11	81.74±8.21	1085/1086	MS, RI
5	Hotrienol	-	-	282.49±47.84	772.76±62.71	1107/1107	MS, RI
6	Geraniol	164.95±27.71	-	324.55±54.96	146.42±0.00	1248/1255	MS, RI
7	(*E*)-2,6-Dimethylocta-3,7-diene-2,6-diol	-	-	343.36±57.75	314.67±23.39	1184/1191	MS, RI
8	*α*-Terpineol	14.71±1.77	6.48±1.40	-	-	1186/1185	MS, RI
9	(*E*)-Nerolidol	8.55±2.35	2.87±0.56	2190.78±353.32	4270.54±717.16	1562/1565	MS, RI
10	Cedrol	1.28±0.47	-	23.19±3.83	28.38±5.11	1598/1596	MS, RI
11	Muurola-4,10(14)-dien-1β-ol	1.40±0.17	1.36±0.00	16.20±0.00	-	1626/1635	MS, RI
12	(*E, E*)-Farnesol	-	-	20.08±3.80	23.43±5.28	1713/1722	MS, RI
13	Isophytol	-	-	10.09±3.53	-	2035/1950	MS, RI
14	Phytol	-	-	279.91±54.15	29.86±13.91	2101/2105	MS, RI
15	*τ*-Muurolol	5.27±1.57	7.07±1.24	87.58±14.60	147.25±28.13	1638/1640	MS, RI
16	*α*-Cadinol	2.3±0.63	4.39±0.89	95.42±23.28	165.56±29.68	1650/1650	MS, RI
17	*n*-Nonadecanol-1	-	-	8.45±4.09	-	2241/2172	MS, RI
18	*D*-Limonene	37.54±5.82	13.28±3.11	-	-	1025/1028	MS, RI
19	*β*-Ocimene	34.55±4.63	9.87±2.78	-	-	1043/1044	MS, RI
20	*γ*-Terpinene	7.66±1.07	-	-	-	1,054/1054	MS, RI
21	allo-Ocimene	19.37±2.87	5.45±1.60	-	55.8±14.23	1124/1131	MS, RI
22	1,3,5,8-Undecatetraene	11.8±2.02	9.57±2.10	-	50.55±0.00	1180/-	MS
23	*α*-Cubebene	-	-	33.28±0.00	50.14±8.72	1345/1345	MS, RI
24	Copaene	2.82±0.21	3.07±0.47	38.02±5.87	60.45±19.80	1371/1367	MS, RI
25	*β*-Cubebene	-	-	-	24.95±5.32	1385/1388	MS, RI
26	*cis*-*α*-Bergamotene	-	-	-	30.27±7.11	1412/1415	MS, RI
27	Caryophyllene	5.82±1.23	3.98±0.6	49.61±8.22	44.9±14.28	1415/1417	MS, RI
28	*cis*-Thujopsene	1.05±0.16	-	-	220.98±0.00	1440/1441	MS, RI
29	(*Z*)-*β*-Farnesene	2.93±0.62	1.4±0.26	109.06±14.79	266.73±43.39	1449/1445	MS, RI

Table S3. (continue)

No.	Volatile Compounds	SFL (ng/g)	WFL (ng/g)	SDT (ng/g)	WDT (ng/g)	RI (cal)/RI (ref)	Qualitative method
30	Aromandendrene	1.02±0.22	1.44±0.40	16.1±2.71	36.82±23.13	1457/1457	MS, RI
31	*γ*-Muurolene	1.11±0.15	1.55±0.56	35.09±6.55	37.3±9.30	1472/1474	MS, RI
32	*α*-Muurolene	2.2±0.18	3.17±0.71	43.95±6.58	3390.27±222.31	1495/1497	MS, RI
33	γ-Cadinene	1.73±0.25	3.08±1.41	30.59±4.87	47.12±12.72	1510/1511	MS, RI
34	*α*-Cadinene	1.41±0.15	1.75±0.27	47.69±6.82	50.89±1.63	1533/1533	MS, RI
35	(*E*)-*α*-Bisabolene	-	-	-	34.98±6.04	1536/1540	MS, RI
36	*α*-Calacorene	2.13±0.28	1.99±0.49	34.95±10.27	33.51±6.84	1538/1542	MS, RI
37	Cadalene	2.48±0.34	1.98±0.37	25.88±5.45	120.07±8.90	1672/1674	MS, RI
38	(+)-*alpha*-curcumene	0.98±0.26	-	30.77±4.75	45.55±7.32	1477/1481	MS, RI
39	8-Heptadecene	0.76±0.23	0.73±0.06	13.30±4.26	19.35±2.84	1668/1670	MS, RI
40	2,2',5,5'-tetramethyl-1,1'-Biphenyl	-	-	-	19.62±0.00	1702/1668	MS, RI
41	1-Octadecene	-	-	9.42±2.21	12.15±2.62	1781/1788	MS, RI
42	Neophytadiene	3.70±1.94	-	199.22±36.99	26.27±6.90	1830/1837	MS, RI
43	*β*-Cyclocitral	4.91±1.39	5.2±1.41	-	80.91±16.72	1216/1218	MS, RI
44	(*E*)-2-Decenal	9.73±2.7	5.56±1.70	-	-	1256/1260	MS, RI
45	Dodecanal	2.35±0.26	4.76±1.69	57.19±9.20	82.75±1.77	1400/1405	MS, RI
46	Benzaldehyde	13.21±1.90	13.57±1.09	124.38±20.73	157.7±12.69	959/961	MS, RI
47	Benzeneacetaldehyde	-	-	311.63±54.32	231.77±44.22	1038/1042	MS, RI
48	(*E*)-2-Hexenal	92.52±17.12	293.85±36.13	-	-	-	MS, RI
49	(*E, E*)-2,4-Heptadienal	-	-	157.87±21.51	109.15±12.93	1007/1007	MS, RI
50	Decanal	31.13±15.11	44.98±17.13	272.31±77.63	177.14±57.21	1199/1206	MS, RI
51	(*E, E*)-2,4-Nonadienal	-	-	40.48±13.10		1208/1199	MS, RI
52	2-butyl-2-Octenal	-	-	32.85±0.89	-	1367/1372	MS, RI
53	2-Dodecenal	-	-	50.98±13.26	-	1458/-	MS
54	Tetradecanal	-	-	15.56±2.39	17.17±0.89	1603/1606	MS, RI
55	Benzoic acid hexyl ester	-	1.08±0.00	244.06±42.26	76.67±18.27	1572/1576	MS, RI

Table S3. (continue)

No.	Volatile Compounds	SFL (ng/g)	WFL (ng/g)	SDT (ng/g)	WDT (ng/g)	RI (cal)/RI (ref)	Qualitative method
56	Diisobutyl phthalate	20.54±17.59	3.09±0.21	14.45±6.47	14.01±8.57	1918/1954	MS, RI
57	Methyl anthranilate	0.91±0.47	0.73±0.12	194.65±34.28	75.76±65.02	1336/1338	MS, RI
58	*β*-Phenylethyl butyrate	-	2.05±0	61.89±9.42	195.03±44.61	1435/1440	MS, RI
59	(*Z*)-3-Hexenyl salicylate	-	-	92.91±20.80	-	1662/1670	MS, RI
60	Methyl jasmonate	0.57±0.11	0.94±0.18	85.83±12.93	498.23±51.81	1642/1644	MS, RI
61	Methyl salicylate	515.85±63.34	156.66±22.33	272.24±45.57	119.90±21.60	1189/1187	MS, RI
62	Hexanoic acid, hexyl ester	4.07±0.60	-	208.30±77.64	74.85±22.24	1378/1383	MS, RI
63	*cis*-3-Hexenyl benzoate	-	-	651.49±107.17	-	1565/1568	MS, RI
64	Isopropyl myristate	-	-	3.89±0.53	5.92±2.58	1815/1823	MS, RI
65	Hexadecanoic acid, methyl ester	7.49±5.93	4.54±0.78	36.77±6.24	22.16±5.57	1954/1939	MS, RI
66	Isopropyl palmitate	0.75±0.10	-	-	6.71±0.65	2042/2023	MS, RI
67	Butylated Hydroxytoluene	1.84±0.51	-	51.55±10.11	52.8±13.66	1506/1513	MS, RI
68	2,4-Di-tert-butylphenol	0.98±0.00	6.00±1.39	-	-	1505/1513	MS, RI
69	Indole	19.16±1.92	9.48±1.83	3020.24±358.27	5453.51±429.69	1291/1293	MS, RI
70	Benzyl nitrile	10.26±1.16	6.2±1.46	775.8±130.11	1335.85±44.98	1134/1143	MS, RI
71	(2-nitroethyl)-Benzene	-	3.23±0.57	166.43±61.59	628.75±81.65	1298/1305	MS, RI
72	Jasmine lactone	-	-	1441.97±209.13	-	1490/1486	MS, RI
73	Dihydroactinidiolide	1.49±0.27	1.54±0.34	88.56±19.02	56.49±7.25	1526/1525	MS, RI
74	Hexanoic acid	56.7±7.69	16.42±3.31	-	202.48±17.54	987/981	MS, RI
75	Geranic acid	-	-	59.74±13.30	-	1349/1355	MS, RI
76	Octanoic acid	-	-	83.05±5.47	37.64±6.00	1172/1175	MS, RI
77	*n*-Decanoic acid	-	1.76±0.00	39.55±6.45	-	1362/1368	MS, RI
78	Myristic acid	-	-	20.04±6.83	15.21±8.90	1751/1759	MS, RI
79	*n*-Hexadecanoic acid	-	-	13.15±5.48	5.27±1.78	2085/1999	MS, RI

Table S3. (continue)

No.	Volatile Compounds	SFL (ng/g)	WFL (ng/g)	SDT (ng/g)	WDT (ng/g)	RI (cal)/RI (ref)	Qualitative method
80	(*Z*)-Jasmone	2.41±0.44	5.97±1.34	469.55±69.20	733.82±87.91	1394/1396	MS, RI
81	*α*-Ionone	0.64±0.25	1.29±0.49	121.14±18.50	21.53±2.93	1421/1427	MS, RI
82	*trans*-*β*-Ionone	8.64±2.03	9.73±1.54	482.04±76.62	255.8±49.25	1481/1485	MS, RI
83	6,10-Dimethyl-5,9-undecadien-2-one	14.38±6.47	20.74±6.68	221.29±41.24	-	1445/1446	MS, RI
84	Hexahydrofarnesyl acetone	3.20±2.88	5.02±2.36	141.23±50.44	52.64±18.31	1835/1838	MS, RI
	Total	1622.20±316.27	1120.32±140.81	16,080.00±2682.96	22,930.00±2687.58		

Table S4. Terpene index of SFL, WFL, SDT and WDT.

	SFL	WFL	SDT	WDT
linalool(ng/g)	75.53	0.00	855.31	1015.55
geraniol(ng/g)	164.95	0.00	324.55	146.42
Terpene index（TI）	0.31	0.00	0.72	0.87

Table S5. The OAV of volatile components in SDT and WDT.

No.	Volatile Compounds	Oder Description	Oder Type	Odor Threshold (ng/g)	OAV	
					SDT	WDT
**1**	(*Z*)-*β*-Farnesene	Floral, citrus[1]	Floral	87[a]	1.25±0.17	3.07±0.50
**2**	Linalool oxide I	Sweet, floral, creamy [2]	Floral	60[b]	6.26±0.97	3.18±0.37
**3**	Linalool oxide II	Sweet, floral, creamy [2]	Floral	100[b]	0.96±0.17	0.82±0.08
**4**	Hotrienol	Fresh, floral, fruity [1]	Floral	110[a]	2.57±0.43	7.03±0.57
**5**	Geraniol	Rose-like, sweet, honey-like[1]	Floral	7.5[a]	43.27±7.33	19.52±0.00
**6**	Indole	Flora[1]	Floral	40[a]	75.51±8.96	136.34±10.74
**7**	(*Z*)-Jasmone	Sweet, floral [1]	Floral	7[a]	67.08±9.89	104.83±12.56
**8**	*α*-Ionone	Floral, violet, powdery, berry-like[1]	Floral	76[a]	1.59±0.24	0.28±0.04
**9**	(*E*)-Nerolidol	Floral, green, citrus, waxy [2]	Floral	10[c]	219.08±35.33	427.05±71.72

Table S5. (continue)

No.	Volatile Compounds	Oder Description	Oder Type	Odor Threshold (ng/g)	OAV	
					SDT	WDT
**10**	(*E, E*)-Farnesol	Floral, sweet, lily-like, muguet-like[1]	Floral	1,000[a]	0.02±0.00	0.02±0.01
**11**	Phytol	Floral [2]	Floral	640[a]	0.44±0.08	0.05±0.02
**12**	Isophytol	Floral, herbal, green [2]	Floral	640[a]	10.09±3.53	——
**13**	Benzeneacetaldehyde	Floral, rose[1]	Floral	4[a]	77.91±13.58	57.94±11.06
**14**	*β*-Phenylethyl butyrate	Sweet, floral[1]	Floral	376[a]	0.16±0.03	0.52±0.12
**15**	Methyl jasmonate	Jasmine-like, floral[1]	Floral	3[a]	28.61±4.31	166.08±17.27
**16**	(*Z*)-3-Hexenyl salicylate	Floral [1]	Floral	1,159[a]	0.08±0.02	——
**17**	Linalool	Sweet, floral [1]	Floral	0.22[a]	3887.77±660.68	4616.14±762.64
**18**	*trans*-*β*-Ionone	Violet, raspberry, flora[1]	Floral	0.01[a]	48,204.00±7662.00	25,580.00±4925.00
**19**	6,10-Dimethyl-5,9-undecadien-2-one	Floral, rose-like [1]	Floral	0.06[b]	3688.17±687.33	——
**20**	Jasmine lactone	Fruity [1]	Fruity	300[e]	4.81±0.70	——
**21**	Benzyl alcohol	Fruity, rose-like [1]	Fruity	20,000[a]	0.01±0.00	0.01±0.00
**22**	Methyl anthranilate	Fruity, concord grape [1]	Fruity	3[a]	64.88±11.43	25.25±21.67
**23**	Methyl salicylate	Peppermint, wintergreen mint [1]	Green	40[a]	6.81±1.14	3.00±0.54
**24**	(*E, E*)-2,4-Nonadienal	green [1]	Green	0.09[c]	449.78±145.56	——
**25**	Decanal	Sweet, citrus, green [1]	Green	0.1[d]	2723.10±776.30	1771.40±572.10
**26**	Benzaldehyde	Caramel, fruity, bitter almond, burnt sugar [1]	Roasted	350[a]	0.36±0.06	0.45±0.04
**27**	*β*-Cyclocitral	Green [1]	Woody	3[a]	——	26.97±5.57
**28**	Caryophyllene	Woody, green, spicy, terpenic [1]	Woody	64[a]	0.78±0.13	0.70±0.22
**29**	Cedrol	Woody，floral [2]	Woody	0.5[c]	46.38±7.66	56.76±10.22
**30**	*α*-Muurolene	Woody [2]	Woody	0.1[b]	439.50±65.80	33,902.70±2,223.10
**31**	(*E, E*)-2,4-Heptadienal	Fatty, green, oily, cinnamon-like [1]	Chemical	0.032[c]	4933.44±672.19	3410.94±404.06

[1] References for odor description citations [1].

[2] References for odor description citations [2].

[a] References for threshold values of volatile compounds citations [2].

[b] References for threshold values of volatile compounds citations [2].

[c] References for threshold values of volatile compounds citations [3].

[d] References for threshold values of volatile compounds citations [4].

[e] References for threshold values of volatile compounds citations [5].

References

1. Guo, X.; Ho, C.; Wan, X.; Zhu, H.; Liu, Q.; Wen, Z. Changes of volatile compounds and odor profiles in Wuyi rock tea during processing. Food Chem. **2021**, 341, 128230.
2. Guo, X.; Ho, C.; Schwab, W.; Wan, X. Effect of the roasting degree on flavor quality of large-leaf yellow tea. Food Chem. **2021**, 347, 129016.
3. Zhai, X.; Zhang, L.; Granvogl, M.; Ho, C.; Wan, X. Flavor of tea (Camellia sinensis): A review on odorants and analytical techniques. Compr. Rev. Food. Sci. Food Saf. **2022**, 21, 3867-3909.
4. Wen, Y.; He, F.; Zhu, B.; Lan, Y.; Pan, Q.; Li, C.; Reeves, M.J.; Wang, J. Free and glycosidically bound aroma compounds in cherry (Prunus avium L.). Food Chem. **2014**, 152, 29-36.
5. Wang, M.; Ma, W.; Shi, J.; Zhu, Y.; Lin, Z.; Lv, H. Characterization of the key aroma compounds in Longjing tea using stir bar sorptive extraction (SBSE) combined with gas chromatography-mass spectrometry (GC–MS), gas chromatography-olfactometry (GC-O), odor activity value (OAV), and aroma recombination. Food Res. Int. **2020**, 130, 108908.
